# Left bundle branch area pacing: A promising modality for cardiac resynchronization therapy

**DOI:** 10.3389/fcvm.2022.901046

**Published:** 2022-11-18

**Authors:** Yuping Fu, Peng Liu, Lingyan Jin, Yingqi Li, Yudi Zhang, Xinghua Qin, Qiangsun Zheng

**Affiliations:** ^1^Department of Cardiology, The Second Affiliated Hospital of Xi’an Jiaotong University, Xi’an, Shaanxi, China; ^2^School of Life Sciences, Northwestern Polytechnical University, Xi’an, Shaanxi, China

**Keywords:** cardiac resynchronization therapy, heart failure, biventricular pacing, conduction system pacing, left bundle branch pacing

## Abstract

Cardiac resynchronization therapy (CRT) is recognized as the first-line management for patients with heart failure (HF) and conduction disorders. As a conventional mode for delivering CRT, biventricular pacing (BVP) improves cardiac function and reduces HF hospitalizations and mortality, but there are still limitations given the high incidence of a lack of response rates. Alternative pacing methods are needed either for primary or rescue therapy. In recent years, conduction system pacing (CSP) has emerged as a more physiological pacing modality for simultaneous stimulation of the ventricles, including His bundle pacing (HBP) and left bundle branch pacing (LBBP). CSP activates the His-Purkinje system, allowing normal ventricular stimulation. However, HBP is technically challenging with a relatively low success rate, high pacing threshold, and failure to correct distal conduction abnormalities. Therefore, LBBP stands out as a novel ideal physiological pacing modality for CRT. Several non-randomized studies compared the feasibility and safety of LBBP with BVP and concluded that LBBP is superior to BVP for delivering CRT with a narrower QRS and greater improvements in left ventricular ejection fraction (LVEF) and New York Heart Association (NYHA) functional class. Concurrently, some studies showed lower and stable pacing thresholds and greater improvement of B-type natriuretic peptide (BNP) levels, as well as better mechanical synchronization and efficiency. LBBP ensures better ventricular electromechanical resynchronization than BVP. In this review, we discuss current knowledge of LBBP, compare LBBP with BVP, and explore the potential of LBBP to serve as an alternative primary therapy to realize cardiac resynchronization.

## Graphical Abstract

**(A)** A photographic representation of LBBP and BVP; **(B)** location of LBBP lead in RAO and LAO 30° view; **(C)** paced ECG after LBBP: a paced RBBB QRS morphology (qR in lead V1) is presented, p-LVAT remains constant and short (80 ms) across different outputs (3, 2.5, and 1.2 V) at the impedance of 670 Ω, but retrograde HIS potential is not recorded; **(D)** advantages of LBBP over BVP. LBBP, left bundle branch pacing; BVP, biventricular pacing; RA, right atrium; LV, left ventricle; RV, right ventricle; CS, coronary sinus; CRT-P, cardiac synchronization therapy-pacemaker; RAO, right anterior oblique; LAO, left anterior oblique; pLVAT, stimulus-to-peak LV activation time; LVEF, left ventricular ejection fraction; HFmrEF, heart failure with mildly reduced ejection fraction. 
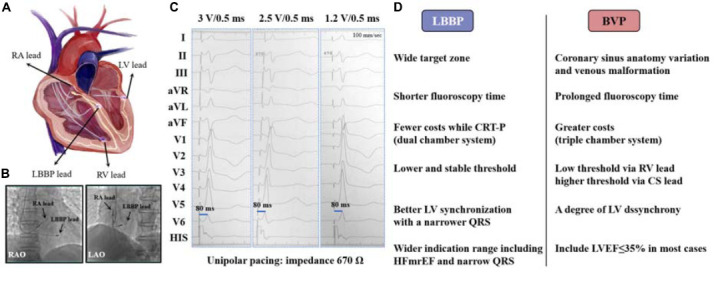

## Introduction

Heart failure (HF) is the terminal state of various cardiovascular diseases that are prone to conduction defects (1), especially the left bundle branch block (LBBB). LBBB can result in ventricular dyssynchrony, which subsequently causes left ventricular contraction dysfunction and HF (2). In addition, long-term right ventricular pacing (RVP), which mimics LBBB, can also lead to pacing-related cardiomyopathy and subsequent HF. According to epidemiological data, approximately one-third of HF patients have a QRS longer than 120 ms, among which 25% have LBBB (3). Therefore, it is of great importance to correct electrical disturbance and ventricular dyssynchrony, especially that caused by LBBB, in HF patients despite the optimized medication options. Since the early 21st century, cardiac resynchronization therapy (CRT) has been recognized as effective non-pharmacological management for moderate to severe HF. Currently, conventional biventricular pacing (BVP) is the first-line therapy for delivering CRT, which improves cardiac function and exercise tolerance and reduces HF-related symptoms, hospitalizations, and mortality by reversing ventricular remodeling (4, 5). However, BVP activates the ventricles non-physiologically, and up to 30–40% of patients do not respond to this pacing method (6). Alternative pacing methods requiring a more physiological mode are needed either for primary or rescue therapy for CRT.

Conduction system pacing (CSP) has gradually attracted public attention in recent years as it directly activates the His-Purkinje system, providing the maximum physiological stimulation to ensure ventricular synchrony, which includes His bundle pacing (HBP) and left bundle branch pacing (LBBP) (7). In 2000, Deshmukh et al. (8) first applied HBP to HF patients with dilated cardiomyopathy and chronic atrial fibrillation (AF). Since then, growing evidence has confirmed the feasibility and safety of HBP in clinical use. In current guidelines, HBP is recommended as a class IIb pacing indication for patients with a mildly reduced left ventricular ejection fraction (LVEF: 36–50%) who need >40% ventricular pacing (9). His capture distal to the intra-Hisian delay site can recruit fibers predestined to be the bundle branches, thereby correcting LBBB and improving HF; this process is also called the “longitudinal dissociation” phenomenon (10). However, the anatomic His bundle area is small and variable and is enfolded by a dense layer of fibrous tissue, making HBP technically challenging with high pacing thresholds and low R wave sensing, as well as disabilities, to correct distal conduction system diseases (11). Moreover, His bundle anatomy increases lead dislocation rates of HBP, and elevated thresholds consequently lead to short battery life, limiting its long-term use (12). In this regard, another novel form of CSP, namely, LBBP stands out. LBBP was initially reported by Huang et al. (13) in 2017 as a rescue pacing therapy for an HF patient with LBBB who failed to achieve both BVP and HBP. Subsequently, LBBP has been expanded and used as a rescue and even a primary strategy for ventricular synchronization in selected patients, including HF patients. Observational studies have shown excellent pacing parameters, narrow QRS duration (QRSd), and improvements in cardiac function by LBBP. However, whether LBBP is superior to BVP for delivering CRT in HF patients remains uncertain. In this review, we will summarize the current knowledge of LBBP, compare it with BVP, and explore the possibility of LBBP as a promising alternative therapy to achieve cardiac resynchronization.

## Novel left bundle branch pacing strategy for cardiac pacing

As more physiological cardiac pacing modalities are in great need to realize cardiac resynchronization, pacing directly through the conduction system has become the focus of attention, especially the novel LBBP with excellent pacing parameters and clinical benefits, which had been gradually put into use in the past 5 years.

### Procedure description

The left bundle branch (LBB) is located underneath the endocardium of the left interventricular septum (IVS) with two main branches, the anterior and the posterior branches, presenting a fan-shaped distribution (14). LBBP is defined as pacing the left bundle trunk or the proximal sites of its branches at the low output (<1.0 V/0.4 ms). Huang et al. (13, 15) first introduced the primary implant technique using Medtronic 3,830 lead [a lumen-less pacing lead (LLL)] and the C315 or C304-His sheath *via* the transseptal way. Routine echocardiography should be performed before implantation to evaluate the thickness of the base IVS as well as the degree of septal scar and fibrosis. in addition, cardiac magnetic resonance imaging (MRI) also presents an available option (16). Twelve-lead surface electrocardiograms (ECGs) and intracardiac electrograms (EGMs) were continuously recorded during the operation. Key steps are described and summarized below.

#### Determining the proper initial site

First of all, the determination of the screwing site on the right side of IVS is a pivotal and initial procedure to endure later success. The 3,830 lead is positioned on the distal His bundle under the right anterior oblique (RAO) view, and the fluoroscopic position is saved as a landmark for identifying the target area unless it is so difficult to locate that the tricuspid annulus is used as a reference. The ideal site to implant LBBP lead is 1.0 to 1.5 cm distal to the saved fluoroscopic lead position, marking the His bundle toward the right ventricular apex on the upper mid-septum under fluoroscopic RAO 30° view (15). Unipolar pacing is performed, and the initial site is identified until a typical “W” pattern with a notch at the nadir of the QRS in lead V1, a positive R waveform in lead II, and an “RS” or “rS” waveform in lead III appear (16). Likewise, discordant aVR/aVL (negative aVR and positive aVL) is used to determine the initial site (17, 18). Recently, another stand stylet-driven pacing lead (SDL) was introduced for LBBP. The process of sheath delivery and lead positioning with SDL is similar to LLL, yet there are still some differences. For example, the lead body of the SDL is wider than that of the LLL due to the presence of an inner lumen, and the SDL is stiffer with the stylet inserted in this lumen (19). Growing evidence supports that LBBP using SDL can achieve a success rate, pacing parameters, and procedural safety comparable to those of LLL (19). However, studies reporting the use of SDL remain limited, and an increasing number of large studies are required to confirm its feasibility. Delivery tools and pacing leads specifically designed for LBBP require further exploration.

#### Penetrating the septum and fixing the lead

Once the initial site is identified, the sheath is rotated counterclockwise to guarantee that the lead tip is perpendicular to the right surface of the septum. Then, the lead is advanced gently toward the left side of the septum with repeated rapid rotation for three to five turns in every attempt to screw it into place. The contrast is injected through the sheath to determine the depth of the lead under a left anterior oblique (LAO) 30° fluoroscopic view (15). Unipolar cathode pacing is intermittently applied during the lead advancement with R wave amplitude, pacing impedance, and paced QRS morphology monitored. Progressive lead advancement results in gradually increasing pacing impedance and an ascending of the notch at the nadir of QRS to form a terminal R wave in lead V1, ultimately presenting a right bundle branch block (RBBB) morphology (a “M” pattern or qR/rsR′) when LBBP is achieved. Unipolar anode pacing was performed to confirm the ring electrode position in the right ventricular septum and estimate the lead depth (18). Septal perforation is one of the most remarkable complications in this process, requiring lead repositioning. To avoid this complication, it is necessary in performing sheath angiography, monitoring QRS morphology, and pacing impedance or monitoring current of injury (COI) during lead advancement (5). A sudden decrease in impedance or sudden disappearance of COI may indicate IVS perforation. Recently, several researchers proposed a fixation beat-guided lead deployment technique, which is defined as premature ventricular contractions (PVCs) with narrow QRS complexes of qR/rsR′ morphology in lead V1. A fixation beat appears when the lead reaches the LBB area and may represent a promising marker for final lead positioning and LBB capture, thus better avoiding septal perforation (20).

#### Confirming the achievement of left bundle branch pacing and sheath removal

To confirm LBB capture, low and high output pacing is performed. Electrical criteria for LBBP are as follows: (1) narrowing of QRS complex (typically ≤130 ms) and a paced RBBB QRS morphology (qR or rSR′ in lead V1); (2) the stimulus-to-peak LV activation time (pLVAT, defined as the interval between pacing stimulus and the peak of R wave in lead V5, V6) remains short (typically <80 ms) and constant regardless of high (5 V) or low (1 V) pacing output; (3) recording LBB potential in patients without LBBB (of note, LBB potential can be recorded only after LBB conduction correction in patients with LBBB); (4) selective (S) or non-selective (NS) LBBP (S-LBBP captures only the LBB with latency from stimulus to QRS and an isoelectric interval, whereas NS-LBBP captures LBB and the adjacent myocardium with no stimulus-QRS latency and isoelectric interval); and (5) recording retrograde His potential or anterograde distal LBB potential (not necessary) (15). Generally, once criterion (1) and at least one of criteria (2) to (5) are achieved, LBB capture is thought to be confirmed, although there is still no consensus. After confirming the LBB capture, further lead advancement is stopped, and the sheath is removed to allow lead slack. Unipolar and bipolar pacing is performed to test the electrical parameters, such as pacing threshold, sensing, and impedance. Resolution of RBBB QRS morphology is seen during unipolar cathode pacing because of retrograde right bundle branch (RBB) activation in some patients. However, in other patients who do not have retrograde RBB stimulation, anodal stimulation (ANS) during bipolar pacing can partially compensate for RBB conduction delay *via* a fusion of LBBP and right ventricular septal capture (21).

### Benefits of left bundle branch pacing in heart failure patients requiring cardiac resynchronization therapy

Heart failure is always accompanied by conduction abnormalities, especially LBBB (3). According to epidemiological data, approximately one-third of HF patients have a QRS longer than 120 ms, among which 25% have LBBB (3). Thus, heart failure with reduced ejection fraction (HFrEF) and LBBB account for the majority of patients requiring CRT (22). Since Huang et al. (13) first applied LBBP in an HF patient with dilated cardiomyopathy and LBBB who failed to achieve both BVP and HBP in 2017, an increasing number of subsequent observational studies have been conducted to explore the feasibility and safety of LBBP in selected patients in need of ventricular pacing. In a prospective study to evaluate the feasibility of permanent LBBP, Li et al. (23) achieved 68.7% (11/16) success in correcting LBBB or RBBB with the paced QRSd narrowed compared with baseline (122.2 ± 9.9 vs. 153.3 ± 27.8 ms). Vijayaraman et al. (24) also found a significantly narrower QRSd from 162 ± 21 to 137 ± 19 ms (*P* < 0.001) after LBBP in HF patients with LBBB who were indicated for CRT [success rate: 88% (21/24)] in their study, whereas QRSd widened from 97 ± 12 to 131 ± 15 ms (*P* < 0.001) in baseline narrow QRS patients, providing a clue for LBBP to realize ventricular electrical resynchronization only if the ventricles were originally asynchronous. Later, increasing evidence merged to support that LBBP benefits ventricular conduction abnormalities. Padala et al. (25) demonstrated narrower paced QRSd compared with baseline (115 ± 12 vs. 144.5 ± 19 ms, *P* < 0.001) by LBBP in patients with the infra-Hisian disease. Similarly, Ravi et al. (26) reported a significantly reduced QRSd *via* LBBP in patients with LBBB (ΔQRSd: 47 ms, *P* < 0.001), RBBB (ΔQRSd: 46 ms, *P* < 0.001), and intraventricular conduction defect (IVCD) (ΔQRSd: 18 ms, *P* = 0.006). Recently, Su et al. (27) conducted a large single-center study with long-term follow-up to evaluate the feasibility of LBBP. Their results similarly revealed a remarkable decrease in paced QRSd in baseline LBBB patients (124.02 ± 24.15 vs. 167.22 ± 18.99 ms, *P* < 0.001) and improvement in LVEF from 48.82 ± 17.78 to 58.12 ± 13.04% (*P* < 0.001) in patients with QRS ≥120 ms. However, most of these studies applying LBBP in bradyarrhythmia indications revealed preserved LVEF or NYHA class but not a significant improvement, which is in contrast with that noted for CRT-indicated patients described in the following part. These studies provide preliminary evidence that LBBP can correct conduction disorders and normalize ventricular electrical synchrony, making it possible to apply LBBP in HF patients requiring CRT.

Several studies were specifically conducted for CRT in HF patients *via* LBBP. Zhang et al. (28) enrolled 11 patients with reduced LVEF and LBBB. They revealed both electrical resynchronization and mechanical resynchronization by LBBP as evidenced by narrowed QRSd (129.09 ± 15.94 vs. 180.00 ± 15.86 ms, *P* < 0.01) and shortened interventricular mechanical delay (IVMD) (14.45 ± 6.38 vs. 61.18 ± 19.46 ms, *P* < 0.0001), respectively. In addition, they observed significant improvement in echocardiographic parameters [LVEF, left ventricular end-systolic diameter (LVESD), *P* < 0.05] and clinical New York Heart Association (NYHA) functional class (*P* < 0.05). Plasma B-type natriuretic peptide (BNP) levels were similarly reduced from 876.00 ± 792.62 to 242.18 ± 267.37 pg/ml (*P* = 0.0067). Huang et al. (29) demonstrated a 97% (61/63) success rate of performing LBBP in HF patients with non-ischemic cardiomyopathy and LBBB. Comparing electrical parameters, LBBP resulted in a pronounced decrease in QRSd (118 ± 12 vs. 169 ± 16 ms, *P* < 0.001) to provide maximum electrical synchrony. At the 1-year follow-up, a significant increase in LVEF (55 ± 10 vs. 33 ± 8%, *P* < 0.001) and a reduction in left ventricular end-systolic volume (LVESV) (67 ± 39 vs. 123 ± 61 ml, *P* < 0.001) were observed. In parallel, LBBP remarkably improved the NYHA class (1.4 ± 0.6 vs. 2.8 ± 0.6, *P* < 0.001) at 1 year. This multicenter study revealed both electric resynchronization and clinical benefits of LBBP for CRT. Similar benefits were demonstrated in the study conducted by Li et al. (30). LBBP shortened QRSd (123.0 ± 10.8 vs. 163.6 ± 29.4 ms, *P* < 0.001), increased LVEF (46.9 ± 10.2 vs. 35.2 ± 7.0%, *P* < 0.001), decreased LV end-diastolic diameter (LVEDD) (56.8 ± 9.7 vs. 64.1 ± 9.9 mm, *P* < 0.001), and improved NYHA class (1.4 ± 0.6 vs. 2.6 ± 0.6, *P* < 0.001) in CRT-indicated patients at a mean follow-up of 9.1 months. In another international, multicenter, collaborative study performed by Vijayaraman et al. (31), LBBP was applied for delivering CRT with an 85% success rate. Notably, a significant QRS narrowing (137 ± 22 vs. 152 ± 32 ms, *P* < 0.01), obvious improvement of LVEF (44 ± 11 vs. 33 ± 10%, *P* < 0.01), and high clinical (72%) and echocardiographic (73%) responses were observed after LBBP. In addition, LBBP showed low and stable pacing thresholds and high R-wave sensing, providing a promising alternative option for delivering CRT in HF patients. Data from Qian et al. (32) evaluating the effects of LBBP in HF caused by chronic RVP also supported these findings. They revealed that LBBP was effective in improving pacing-induced HF by improving cardiac function (LVEF increasing: 48.1 ± 9.5 vs. 40.3 ± 5.2%, *P* = 0.002), improving NYHA class (1.7 ± 0.8 vs. 2.5 ± 0.5, *P* < 0.0001), decreasing N-terminal pro-brain natriuretic peptide (NT-proBNP) levels (1,840 ± 2,261 vs. 3,178 ± 2,974 pg/ml, *P* = 0.005), and normalizing electrical synchrony (QRSd narrowing: 116.6 ± 11.7 vs. 174.1 ± 15.8 ms, *P* < 0.0001). No lead-related complications occurred in this study. In elderly patients who are typically precluded from BVP due to their frailty, LBBP seems to be a better option with a lower risk of CRT complications. Grieco et al. evaluated the feasibility and safety of LBBP-CRT in elderly patients. The study compared electrical parameters, echocardiographic parameters, lead parameters, and complications, revealing that LBBP achieved comparable efficacy between elderly patients and younger patients with narrow QRS, satisfactory pacing threshold, impedance and sensing, low rates of complication, and improved LVEF (33). In summary, LBBP is a promising option for delivering CRT in HF patients, providing excellent ventricular resynchronization, improved cardiac function, and great clinical benefits with a high success rate, low and stable pacing thresholds, and fewer complications.

## Comparison of left bundle branch pacing with biventricular pacing as a treatment for heart failure

### Current studies comparing left bundle branch pacing with biventricular pacing

Biventricular pacing has long been a standard method for delivering CRT in symptomatic HF patients for approximately 20 years, yet LBBP has recently emerged as a promising alternative modality. Several non-randomized observational studies have shown the advantages of LBBP over conventional BVP by comparing electrical parameters, echocardiographic parameters, and clinical outcomes. These results are summarized in Table 1. Li et al. (34) prospectively compared the efficacy of LBBP and BVP in HF patients during a 6-month follow-up period. The results showed that LBBP required less X-ray exposure time (16.9 ± 6.4 vs. 39.6 ± 9.2 min, *P* < 0.001) than BVP at the implant, indicating better safety for the operator and patients. Their results also revealed a much more significant decrease in paced QRSd (58.0 vs. 12.5 ms, *P* < 0.001) in the LBBP group than in the BVP group, as well as enhanced LVEF improvement (15.6 vs. 7.0%, *P* < 0.001) during the 6-month follow-up. Another non-randomized study performed by Wu et al. (35) recruited CRT-indicated patients for BVP, HBP, or LBBP. In this study, BVP or HBP was applied as the primary therapy, whereas LBBP was used as rescue therapy for HBP-failed patients. The paced QRSd was significantly decreased both in the LBBP group (ΔQRSd = 56 ms, *P* < 0.001) and the BVP group (ΔQRSd = 26 ms, *P* < 0.001). A comparison of LBBP with BVP was not shown, although there was a trend toward a greater reduction in QRSd in the LBBP group. Echocardiographic benefits were greater in the LBBP group than in the BVP group, as evidenced by greater LVEF improvement (ΔLVEF) in the LBBP group than in the BVP group (24.0 ± 10.9 vs. 16.7 ± 14.6%, *P* = 0.015) and a considerably improved echocardiographic response in the LBBP group than in the BVP group (ΔLVEF ≥10%, 93.3 vs. 61.2%, *P* = 0.004; ΔLVEF ≥15%, 76.7 vs. 53.1%, *P* = 0.036; final LVEF ≥50%, 70.0 vs. 44.9, *P* = 0.030) at the 1-year follow-up. LBBP also resulted in greater improvement in NYHA class (*P* = 0.002) and a trend toward greater improvement in BNP levels (*P* = 0.099) compared with the BVP group, indicating a better clinical response. Supportively, the BVP group exhibited a higher adverse event rate. Specifically, three patients experienced HF hospitalization and two patients were transferred to HBP. In contrast, none of these events occurred in the LBBP group. Similarly, Wang et al. (36) compared the efficacy of LBBP with BVP in HF patients with complete LBBB. LBBP shortened QRSd much more than BVP (60.80 ± 20.09 vs. 33.00 ± 21.48 ms, *P* = 0.0009). The echo-LVEF exhibited better improvements in the LBBP group than in the BVP group, although the difference was not statistically significant (*P* = 0.11). Changes in clinical NYHA class were significant, and more patients were classified as NYHA I/II in the LBBP group than in the BVP group (median 1.5 vs. 2.0, *P* = 0.029) at the 6-month follow-up. Consistently, Guo et al. (37) demonstrated a greater QRSd reduction (56.0 ± 14.7 vs. 32.3 ± 14.6 ms, *P* < 0.0001) in the LBBP group than in the BVP group, whereas no significant difference was observed in echocardiographic parameters or clinical NYHA class change (although there was a trend toward better improvement *via* LBBP), which may be due to a relatively small sample size. Advantages in LBBP were noted compared with BVP with lower and stable pacing thresholds (0.48 ± 0.22 V at 0.4 ms vs. 1.12 ± 0.46 V at 0.4 ms, *P* < 0.0001). Recently, Zu et al. (38) evaluated and compared the feasibility of LBBP with BVP for delivering CRT. Shorter operation time (90.08 ± 33.40 vs. 158.05 ± 19.05 min, *P* < 0.01) and X-ray exposure time (20.46 ± 7.36 vs. 43.53 ± 10.36 min, *P* < 0.01) were achieved in the LBBP group. Better electrical resynchronization was observed in the LBBP group as evidenced by greater QRS shortening (50.30 ± 23.79 vs. 33.15 ± 20.22 ms, *P* = 0.036) compared with the BVP group. At the 12-month follow-up, LVEF improved more in the LBBP group compared with the BVP group (48.92 ± 8.06 vs. 42.53 ± 4.89%, *P* < 0.05). The increased safety and feasibility of LBBP compared with BVP were further confirmed in a multicenter study conducted by Chen et al. (39) in HF patients with LBBB. Their results showed a high success rate in both groups (98.00% in the LBBP group and 91.07% in the BVP group). The LBBP group exhibited a shortened QRSd as compared to the BVP group (102.61 ± 9.66 vs. 126.54 ± 11.67 ms, *P* < 0.001) and better improvement in LVEF both during the 6-month (47.58 ± 12.02 vs. 41.24 ± 10.56%, *P* = 0.008) and 1-year follow-ups (49.10 ± 10.43 vs. 43.62 ± 11.33%, *P* = 0.021). A stable and lower pacing threshold was observed in the LBBP group both at implant (*P* < 0.001) and the 1-year follow-up (*P* < 0.001). Adverse clinical outcomes and complications were comparable in the LBBP and BVP groups. The studies above evaluated left ventricular electrical synchrony; however, mechanical resynchronization of LBBP was less explored and compared. In a multicenter, prospective cohort study, Liu et al. (40) specifically evaluated the mechanical synchrony of LBBP and compared it with that of BVP. HF patients with complete LBBB who underwent LBBP or BVP were enrolled in the study. Compared with BVP, LBBP resulted in better QRS shortening (64.1 ± 18.9 vs. 32.5 ± 22.3 ms, *P* < 0.001). The interventricular and intraventricular mechanical synchronization, reflected by IVMD and peak strain dispersion (PSD), respectively, were better improved in the LBBP group compared with the BVP group (ΔIVMD: 27.4 ± 28.7 vs. 18.6 ± 27.9 ms, *P* = 0.013; ΔPSD: 50.9 ± 56.8 vs. 26.9 ± 63.9 ms, *P* = 0.036). Moreover, global and segmental myocardial work were evaluated in both groups. Global work efficiency (GWE), global work index (GWI), and global constructive work (GCW) were better improved in the LBBP group compared with the BVP group (*P* < 0.05). For each LV segment myocardial work, LBBP showed more improvements in most segments than in the BVP group, especially the lateral (*P* = 0.006) and posterior (*P* = 0.068) segments. Taking all these studies together, we hypothesize better electrical resynchronization, mechanical synchrony, and clinical benefits of LBBP in HF patients requiring CRT than conventional BVP. Furthermore, we searched studies comparing LBBP with BVP in HF patients with AF and narrow QRS in need of atrioventricular node ablation. Ivanovski et al. (41) found shorter-paced QRSd in the LBBP group compared with the BVP group (127.0 ± 13.0 vs. 172.0 ± 13.0 ms, *P* < 0.001), whereas no significant difference in baseline QRSd was noted. Echocardiographic results showed improved LVEF (*P* = 0.041) and decreased indexed LV volumes (*P* = 0.004) in the LBBP group during follow-up, but no significant change was observed in the BVP group (*P* = 0.916 for LVEF; *P* = 0.551 for indexed LV volumes). Regarding clinical outcomes, the follow-up NYHA class did not differ from the baseline NYHA class in the BVP group (*P* = 0.096). However, in the LBBP group, NYHA class was significantly improved at the 6-month follow-up (*P* = 0.008). NT-proBNP levels were reduced at follow-up in the LBBP group (*P* = 0.047) but remained unchanged in the BVP group (*P* = 0.331). Therefore, LBBP is not only beneficial for HF patients requiring CRT with wide QRS but also provides superior electrical, symptomatic, and echocardiographic improvements than BVP in HF patients with AF and narrow QRS who require atrioventricular node ablation. Recently, the first prospective, randomized trial performed by Wang et al. compared LBBP with BVP for CRT (42). Consistent with the observational studies above, LBBP showed higher LVEF improvement than BVP (21.08 ± 1.91 vs. 15.62 ± 1.94%, *P* = 0.039) and a trend toward a greater decrease in LVESV (77.74 ± 7.80 vs. 55.58 ± 8.80 ml) and NT-proBNP (1,768.36 ± 217.91 vs. 1,181.05 ± 216.75 pg/ml). However, there were comparable changes in QRSd, NYHA class, and the 6-min walk distance between the two groups.

**TABLE 1:** Studies comparing LBBP with BVP.

References	Design	*N*	Success rate of LBBP	Rescue LBBP (*n*)	Cross-over from CSP to BVP (*n*)	Criteria of inclusion/ exclusion	Patients with AVB	Follow-up (m)	Pacing parameter	Electrical or mechanical changes	Echocardiographic changes	Clinical changes
Li et al. (34)	Prospective, multicenter, observational	27 vs. 54	73.0% (27/37)	9	4	Inclusion: HF symptoms, LVEF ≤35% with LBBB	Not mentioned	6	Threshold: 0.81 vs. 1.22 V Impedance: 644.9 vs. 817.5 Ω	Baseline QRSd: 178.2 ± 18.8 vs. 180.9 ± 29.7 ms Paced QRSd: 121.8 ± 10.8 vs. 158.2 ± 21.5 ms ΔQRSd: 58.0 vs. 12.5 ms Baseline QRS morphology: LBBB	Baseline LVEF: 28.8 ± 4.5 vs. 27.2 ± 4.9% Follow-up LVEF: 44.3 ± 8.7 vs. 35.0 ± 10.5% ΔLVEF: 15.6 vs. 7.0% ΔLVEDD: 8.0 vs. 0.5 mm Echocardiographic response: 88.9 vs. 66.7% Super response: 44.4 vs. 16.7%	Baseline NYHA: 3.1 ± 0.7 vs. 3.0 ± 0.7 Follow-up NYHA: 1.5 ± 0.5 vs. 2.3 ± 0.7 clinical response: 96.3 vs. 75.9%
Wu et al. (35)	Prospective, non-randomized, single-center	32 vs. 54	100% (32/32)	32	15	Inclusion: LVEF ≤40% and typical LBBB	3.1 vs. 3.7%	12	Threshold: 0.49 (LBBP) vs. 0.61 (RV lead)/0.93 V (CS lead) Sensing: 11.2 vs. 14.1 mV	Baseline QRSd: 166.2 ± 16.2 vs. 161.1 ± 18.2 ms Paced QRSd: 110.8 ± 11.1 vs. 135.4 ± 20.2 ms ΔQRSd: 56.0 vs. 26.0 ms Baseline QRS morphology: LBBB	Baseline LVEF: 30.4 ± 7.1 vs. 29.7 ± 5.1% Follow-up LVEF: 54.4 ± 9.8 vs. 46.5 ± 16.9% ΔLVEF: 24.0 vs. 16.7% LVESV: 54.6 vs. 84.8 ml	Baseline NYHA: 2.8 ± 0.5 vs. 2.8 ± 0.6 Follow-up NYHA: 1.3 ± 0.5 vs. 1.9 ± 0.9
Wang et al. (36)	Matched case–control study	10 vs. 30	100% (10/10)	0	0	Inclusion: HF, LBBB with QRSd >140 ms in men and >130 ms in women, LVEF ≤35%, and NYHA II to IV	Not mentioned	6	Threshold: 0.54 vs. 1.00 V	Baseline QRSd: 183.60 ± 19.27 vs. 174.60 ± 19.48 ms Paced QRSd: 122.80 ± 17.24 vs. 141.60 ± 15.38 ms ΔQRSd: 60.8 vs. 33.0 ms Baseline QRS morphology: LBBB	Baseline LVEF: 26.80 ± 3.85 vs. 26.38 ± 5.27% Follow-up LVEF: 45.66 ± 9.22 vs. 39.35 ± 12.29% ΔLVEF: 18.86 vs. 12.97% Response rate: 100.00 vs. 63.33%	Baseline NYHA: 2.90 ± 0.74 vs. 3.07 ± 0.74 Follow-up NYHA: 1.50 ± 0.55 vs. 1.97 ± 0.61
Guo et al. (37)	Prospective, observational	21 vs. 21	87.5% (21/24)	0	3	Inclusion: HF, LBBB morphology, with LVEF ≤35%, NYHA II to IV	Not mentioned	6	Threshold: 0.48 (LBBP) vs. 0.57 (RV lead)/1.12 V (CS lead)	Baseline QRSd: 167.7 ± 14.9 vs. 163.6 ± 13.8 ms Paced QRSd: 111.7 ± 12.3 vs. 130.1 ± 14.0 ms ΔQRSd: 56.0 vs. 32.3 ms Baseline QRS morphology: LBBB	Baseline LVEF: 30.0 ± 5.0 vs. 29.8 ± 4.1% Follow-up LVEF: 50.9 ± 10.7 vs. 44.4 ± 13.3% LVEF: 50.9 vs. 44.4% Super response: 80.9 vs. 57.1%	Baseline NYHA: 3.0 ± 0.7 vs. 3.0 ± 0.7 Follow-up NYHA: 1.3 ± 0.9 vs. 1.5 ± 0.7
Zu et al. (38)	Observational	13 vs. 19	100% (13/13)	3	0	Inclusion: DCM complicated with HF and LBBB, ischemic cardiomyopathy was excluded	30.8 vs. 10.5%	12	Comparison not mentioned	Baseline QRSd: 167.46 ± 28.11 vs. 163.47 ± 21.66 ms Paced QRSd: 117.15 ± 9.91 vs. 130.32 ± 12.41 ms ΔQRSd: 50.30 vs. 33.15 ms Baseline QRS morphology: LBBB	Baseline LVEF: 30.62 ± 6.983 vs. 29.11 ± 4.818% Follow-up LVEF: 48.92 ± 8.06 vs. 42.53 ± 4.89%	Not mentioned
Chen et al. (39)	Prospective, multi-center, observational	49 vs. 51	98.0% (49/50)	5	1	Inclusion: HF, NYHA II–IV, LVEF ≤35%, QRSd >150 ms, typical LBBB Exclusion: PR interval >200 ms, persistent AF and IVCD	Not mentioned	12	Threshold: 0.92 vs. 1.45 V	Baseline QRSd: 180.12 ± 15.79 vs. 175.70 ± 11.29 ms Paced QRSd: 102.61 ± 9.66 vs. 126.54 ± 11.67 ms ΔQRSd: 59.16 vs. 31.00 ms Baseline QRS morphology: LBBB	Baseline LVEF: 29.05 ± 5.09 vs. 28.36 ± 5.30% Follow-up LVEF: 49.10 ± 10.43 vs. 43.62 ± 11.33% ΔLVEF: 20.9 vs. 15.2% LVEDD: 54.50 vs. 60.99 mm LVESD: 41.78 vs. 48.33 mm Super response: 61.22 vs. 39.22%	Baseline NYHA (percentage of III–IV): 91.48 vs. 88.24% Follow-up NYHA (percentage of III–IV): 4.08 vs. 19.61%
Liu et al. (40)	Prospective, multicenter, cohort study	27 vs. 35	79.4% (27/34)	0	7	Inclusion: HF, LVEF ≤35%, LBBB morphology and QRSd ≥130 ms Exclusion: narrow QRS or non-LBBB morphology	Not mentioned	3–6	Not mentioned	Baseline QRSd: 177.1 ± 16.7 vs. 168.8 ± 16.8 ms Paced QRSd: 113.0 ± 18.4 vs. 136.3 ± 20.1 ms ΔQRSd: 64.1 vs. 32.5 ms Baseline QRS morphology: LBBB Better mechanical synchrony reflected by IVMD, PSD, GWE, GWI, GCW, MWE	Baseline LVEF: 29.9 ± 4.8 vs. 29.5 ± 4.9% Follow-up LVEF: 47.1 ± 8.3 vs. 43.1 ± 11.0% ΔLVEF: 17.2 ± 9.3 vs. 13.7 ± 11.5% Echocardiographic response: 88.9 vs. 68.6%	Baseline NYHA: 3.0 ± 0.5 vs. 2.8 ± 0.6 Follow-up NYHA: 1.6 ± 0.6 vs. 2.2 ± 0.8 ΔNYHA: 1.6 ± 0.6 vs. 0.9 ± 0.8
Ivanovski et al. (41)	Retrospective, single-center, observational	10 vs. 13	100% (10/10)	0	0	Inclusion: severely symptom AF with rapid ventricular rate, tachycardia-induced cardiomyopathy, LVEF <50%, NYHA II–IV, narrow QRSd ≤120 ms	Not mentioned	6	Threshold: 0.80 vs. 1.40 V Impedance: 749.0 vs. 760.0 Ω	Baseline QRSd: 105 ± 15 vs. 98 ± 7 ms Paced QRSd: 127 ± 13 vs. 172 ± 13 ms ΔQRSd: −29.0 vs. −74.0 ms Baseline QRS morphology: LBBB	Baseline LVEF: 28.0 vs. 38.0% Follow-up LVEF: 40.0 vs. 37.0% ΔLVEF: 12.0% vs. −1.0%	Baseline median NYHA: 3.0 vs. 3.0 Follow-up median NYHA: 2.0 vs. 3.0 ΔNT-proBNP: 1,057.0 vs. 52.0 pg/ml
Wang et al. (42)	Prospective, randomized trial	22 vs. 18	91.7% (22/24)	4	2	Inclusion: age 18–80 years, sinus rhythm, complete LBBB meeting Strauss’s standard definition (QRSd >140 ms for men or >130 ms for women), LVEF ≤40%, and NYHA class II to IV Exclusion: (1) ischemic cardiomyopathy; (2) non-LBBB QRS morphology including RBBB or IVCD; (3) persistent AF; or (4) pregnancy	Not mentioned	6	Threshold: 0.82 vs. 1.12 V Impedance: 476.0 vs. 592.0 Ω	Baseline QRSd: 174.6 ± 14.3 vs. 174.7 ± 14.1 ms Paced QRSd: 131.5 ± 12.5 vs. 136.6 ± 12.9 ms Baseline QRS morphology: LBBB	Baseline LVEF: 28.3 ± 5.3 vs. 31.1 ± 5.6% Follow-up LVEF: 49.4 ± 13.2 vs. 46.5 ± 9.4% ΔLVEF: 21.08 ± 1.91 vs. 15.62 ± 1.94% Super response: 65.0 vs. 42.1%	Baseline NYHA: 2.40 ± 0.50 vs. 2.45 ± 0.51 ΔNYHA: 1.22 ± 0.11 vs. 1.10 ± 0.11 Δ6-min walk distance: 100.69 ± 14.14 vs. 80.56 ± 15.92 m ΔNT-proBNP: 1,768.36 ± 217.91 vs. 1,181.05 ± 216.75 pg/ml

LBBB, left bundle branch block; LBBP, left bundle branch pacing; BVP, biventricular pacing; CSP, conduction system pacing; AVB, atrioventricular block; IVCD, intraventricular conduction defect; AF, atrial fibrillation; LV, left ventricle; RV, right ventricle; CS, coronary sinus; Δ, change of parameters; QRSd, QRS duration; LVEF, left ventricular ejection fraction; LVEDD, left ventricular end-diastolic diameter; LVESD, left ventricular end-systolic diameter; LVESV, left ventricular end-systolic volume; IVMD, interventricular mechanical delay; PSD, peak strain dispersion; GWE, global work efficiency; GWI, global work index; GCW, global constructive work; MWE, myocardial work efficiency; NYHA, New York Heart Association. NT-proBNP, N-terminal pro-brain natriuretic peptide. All the numerical values ahead of “vs.” represents the LBBP group while numerical values that comes after “vs.” represents the BVP group. The symbol “−” in the “Electrical or mechanical” column represents an increase in QRSd.

### Clinical limitations of biventricular pacing for heart failure management

Despite the long history of the use of BVP as a standard CRT modality, some challenges continue to emerge regarding the growing need for a more ideal cardiac pacing strategy. First of all, BVP is a non-physiological pacing mode unable to realize effective ventricular synchronization due to epicardial LV pacing, which forces the ventricle to depolarize from the epicardium to the endocardium, in contrast to the physiologic way and predisposes to torsade de pointes tachycardias (43, 44). In addition, long-term RVP does not meaningfully improve RV systolic function and even worsens RV asynchrony, thus causing RV remodeling and aggravating HF or leading to ventricular arrhythmias, including AF (45). Approximately 30–40% of patients remain non-responsive to BVP due to ischemic heart disease, remarkable left atrial dilation, advanced mitral regurgitation, and NYHA class IV. (46). In addition, coronary sinus anatomy and venous malformation limit the intravenous implantation of epicardial LV leads (47) despite improvements in delivery leads and tools and refinements in implant technique. Transvenous LV lead implantation also leads to a chronic increase in the capture threshold, a high lead dislocation rate (up to 5–10%) (48), and phrenic nerve stimulation. Furthermore, BVP fails to stimulate myocardial scar or severely diseased myocardium (4). To address some of these issues, several new target approaches, such as multipoint LV pacing and endocardial LV pacing, have been proposed. Multipoint LV pacing is realized by a multipolar (typically quadripolar) LV lead which is positioned from the coronary sinus branch (49). Three ring electrodes are located at 20, 30, and 47 mm from the tip (50) to achieve multi-site pacing, which can better avoid tissue scar, thereby achieving better electrical synchrony than conventional BVP (51). The Multi-Point Pacing Study (52) and the LV Multi-spot Pacing for CRT (iSPOT) study (53) revealed the non-inferiority of multipoint pacing compared with conventional BVP. However, the feasibility of multipoint pacing remains uncertain, and battery longevity is reduced (6, 54). Moreover, still positioning from the coronary sinus branch as BVP (49), multipoint LV pacing is essentially a type of epicardial LV pacing and thus not an ideal physiological pacing modality. Endocardial LV pacing through a transseptal approach delivers a more rapid and physiological stimulation of LV eliminating phrenic nerve stimulation compared with conventional epicardial LV pacing (55), and it has been proven in the ALSYNC (Alternate Site Cardiac Resynchronization) study to be non-inferior and even superior to conventional BVP with improved hemodynamic response and clinical outcomes even noted in conventional BVP non-responders (56). However, the thromboembolic event rate in this study remained high despite the use of anticoagulants (57). Later, leadless LV pacing was developed to address some of the lead-related complications, which are still under research. Using this method, LV pacing is achieved wirelessly by converting acoustic energy to pacing energy with a receiving electrode in the LV wall (58). A meta-analysis conducted recently concluded that leadless LV pacing could serve as a second-line therapy for conventional CRT non-responders (59). In recent years, BVP has been expanded from severe HF (NYHA III/IV) to mild to moderate HF (NYHA I/II), yet HF patients with a narrow QRS (<130 ms) do not respond to it or benefit from it in most cases. BVP is recommended only as a class III, level A indication in the 2021 ESC guidelines (55). Moreover, CRT-indicated patients are typically those with LVEF ≤35%, but rare patients with LVEF >35% [heart failure with mildly reduced ejection fraction (HFmrEF)] are indicated for conventional CRT or benefit from it. In the 2018 ACC/AHA/HRS guidelines, HF patients with mild to moderate reduced LVEF (36–50%) are recommended as a class IIb, and level C CRT indication only if they have LBBB (QRS ≥150 ms) (9). These limitations above restrain the clinical use of BVP for HF or expanded patients.

### Potential advantages of left bundle branch pacing over biventricular pacing

Left bundle branch area pacing (LBBAP) may partially solve some of the obstacles mentioned above, including left ventricular septal pacing (LVSP) and LBBP. LVSP is defined by pacing at the left side of the interventricular septum (IVS), which was first proposed by Mafi-Rad et al. (60). Later, LVSP showed acute hemodynamic and electrophysiological benefits equal to BVP in a study performed by Salden et al. (61), but the long-term clinical outcomes have not been investigated. The difference between LBBP and LVSP is whether they capture the LBB (only LBBP does) (62). However, there areno definite criteria for LVSP. Both pacing modes provide nearly physiological LV activation (63) by endocardial pacing and stimulating the left side of the IVS first. LBBP activates the ventricles more physiologically than LVSP by directly stimulating the conduction system, resulting in a shorter pLVAT (64). Emerging studies pay attention to the clinical use of LBBP, and the potential advantages of LBBP compared with conventional BVP are listed in Table 2. First, LBBP provides physiological LV activation starting at the IVS, from the endocardium to the epicardium, which provides maximum ventricular synchronization with a narrower QRS (65). Given that electrical synchronization is achieved more effectively, LBBP achieves considerably improved ventricular mechanical synchrony than BVP to ensure normal electromechanical coupling of the myocardium. The success rates of LBBP (85–100%) (4) and BVP (85–95%) (4) are comparable, yet approximately 30% of patients do not respond to BVP (6, 11). Advancing the lead *via* the transseptal approach, implantation of LBBP is rarely influenced by the anatomic variation of the vessels, making it easier to perform with high success rates. In addition, the procedure is safer for the operators and patients given the short procedure time and X-ray exposure time. Although septal perforation, thromboembolism, lead dislodgement, and other complications might probably occur (17), previous studies (23, 25, 27, 66) reported few complication events, and these events could be avoided or reduced by cautious operation with rare long-term damage. Relatively speaking, LBBP has a lower and more stable capture threshold during the procedure and in long-term follow-up, whereas fewer lead dislocation events are noted compared with BVP. Based on its application in patients with HFmrEF in several studies (29–32), LBBP is considered effective in an expanded group of patients for wider indications. In addition, LBBP has also been used in HF patients with narrow QRS and AF in need of atrioventricular node ablation, suggesting its benefits despite QRSd and providing a wider indication for the application of LBBP in HF patients. Moreover, in elderly patients who are typically precluded from BVP due to CRT complications, LBBP seems to represent a better option. Nevertheless, in scenarios in which patients do not respond to BVP, such as ischemic heart disease, marked left atrial dilation, advanced mitral regurgitation, and severely diseased myocardium, LBBP may not serve as an ideal pacing strategy to solve these obstacles either. Moreover, delivery tools and leads of LBBP are quite limited, and leads designed for HBP are currently being used.

**TABLE 2:** Comparison of advantages and disadvantages between LBBP and BVP.

	LBBP	BVP
Anatomy	Wide target zone underneath the endocardium of left side of IVS	Coronary sinus anatomy variation and venous malformation limits LV lead implantation
Safety	Safer for operators and patients with shorter operation and fluoroscopy time	Prolonged operation and X-ray exposure time
Costs	Fewer costs because of a dual chamber system in CRT-P, yet comparable costs with BVP in scenario that needs a CRT-D	Greater costs for a triple chamber system
Technical difficulty	Relatively easier	A little more difficult due to various coronary sinus anatomy
Delivery tools and leads	Limited and still using leads designed for HBP	Numerous as endocardial LV pacing, multi-point LV pacing developing
Success rate	85–100% (4)	85–95% (4)
Respond rate	Not clear	Around 70% (6, 11)
Pacing parameters	Lower and stable threshold, high R wave sensing	Higher threshold *via* CS lead
Cardiac synchrony	Better electromechanical synchronization with a narrower QRS	A degree of LV dyssynchrony because of non-physiological pacing with a wider QRS
Indication range	Wider, including HFmrEF and HF with narrow QRS such as AF patients with atrioventricular node ablation	Narrower, with wide QRS (≥130 ms) and usually those whose LVEF ≤35% in most cases
Complications	Comparable, septal perforation	Comparable, phrenic nerve stimulation

LBBP, left bundle branch pacing; BVP, biventricular pacing; CRT-P, cardiac resynchronization therapy-pacemaker; CRT-D, cardiac resynchronization therapy-defibrillator; HBP, his bundle pacing; LV, left ventricle; RV, right ventricle; CS, coronary sinus; IVS, interventricular septum; LVEF, left ventricular ejection fraction; HFmrEF, heart failure with mildly reduced ejection fraction.

### Left bundle branch area pacing-optimized cardiac resynchronization therapy for severe heart failure

In patients with more advanced HF, severe electrical asynchrony may exist such as distal conduction delay and IVCDs. In these scenarios, LBBAP alone may not achieve ideal ventricular electrical synchronization. Instead, BVP can partially offset those conduction abnormalities. Therefore, LBBAP combined with BVP (sequential LV pacing) might provide better synchronization and clinical outcomes in these patients. Jastrzebski et al. (67) conducted a prospective observational multicenter study for left bundle branch area pacing-optimized cardiac resynchronization therapy (LOT-CRT) in CRT-indicated patients or non-responders to BVP alone. Patients recruited had severe ventricular dyssynchrony with a wide baseline QRSd of 181 ± 26 ms. Greater QRS narrowing *via* LOT-CRT was noted compared with either BVP or LBBAP alone (144 ± 22 vs. 170 ± 30 vs. 162 ± 23 ms, *P* < 0.0001), suggesting better electrical synchrony when these two approaches are combined for selected patients. For echocardiographic benefits, LOT-CRT improved LVEF (37.2 ± 12 vs. 28.5 ± 9.9%, *P* < 0.0001) and decreased left ventricular end-diastolic volume (LVEDV, 171.4 ± 83 vs. 209.8 ± 99, *P* < 0.0001) and LVESV (110.6 ± 69 vs. 149.5 ± 84, *P* < 0.0001) compared to baseline. Furthermore, the NYHA class was improved from 2.9 ± 0.6 to 1.9 ± 0.6 (*P* < 0.0001), and serum NT-proBNP levels decreased from 5,668 ± 8,249 pg/ml to 2,561 ± 3,555 pg/ml (*P* < 0.0001) during the 3-month follow-up. In subgroup analysis, using LBBP for LBBAP provided a greater reduction of QRSd (141 ± 20 vs. 152 ± 25 ms, *P* = 0.028) and greater improvement of LVEF (ΔLVEF: 11.1 ± 11.3 vs. 4.7 ± 7.5, *P* = 0.0196) than LVSP, indicating the advantages of the LBBP-BVP combined LOT-CRT strategy compared with LVSP-BVP strategies.

## Limitations and future directions

To date, BVP remains the standard CRT option supported by evidence-based medicine and recommended in guidelines. However, LBBP has quickly developed and spread, especially in China, during the last 5 years. LBBP still serves as a rescue therapy for BVP-failed patients and is not routinely used in clinical practice. Long-term safety and complications, including arrhythmias, require further exploration in large-scale random controlled trials. More prospective, large-scale studies comparing LBBP with conventional BVP with a longer follow-up period and definite clinical endpoints are required to decide which pacing modality is superior for HF patients. Obstacles in determining precise indications for LBBP or BVP must be solved perhaps by recruiting more HF patients who have ischemic cardiomyopathy or non-specific IVCDs, not only typical LBBB. Moreover, LBBP alone is less likely to achieve ideal electrical synchrony in patients with severe electrical dyssynchrony such as IVCD, whereas BVP can partially solve these problems. Thus, LOT-CRT combining LBBP and BVP is required in the treatment of more advanced HF in future, and more studies involving LOT-CRT should be conducted to further verify its clinical efficacy and long-term safety. Finally, improvements or special designs in delivery leads, sheaths, and devices for better adaptation to LBBP are also a matter of concern given that a set of delivery tools suitable for HBP is continuously used in LBBP currently.

## Conclusion

Left bundle branch pacing represents a novel CSP modality that demonstrates promise for replacing the standard application of conventional BVP for CRT in HF patients in future, offering physiological activation of the ventricles. Advantages of LBBP have emerged in recent years over BVP, including better ventricular electrical and mechanical resynchronization and improvements in cardiac function, NYHA function class, and clinical outcomes. Further prospective randomized trials involving larger populations are required to provide further evidence for the safety and feasibility of LBBP and expand its primary clinical use.

## Author contributions

QZ and XQ provided the idea and technical guidance and revised the manuscript. YF wrote the manuscript. PL, LJ, YL, and YZ made the tables and figure. All authors reviewed and approved the manuscript.

## Abbreviations

AF: atrial fibrillation
ANS: anodal stimulation
BNP: B-type natriuretic peptide
BVP: biventricular pacing
COI: current of injury
CRT: cardiac resynchronization therapy
CRT-D: cardiac resynchronization therapy-defibrillator
CRT-P: cardiac resynchronization therapy-pacemaker
CSP: conduction system pacing
ECGs: electrocardiograms
EGMs: electrograms
GCW: global constructive work
GWE: global work efficiency
GWI: global work index
HBP: His bundle pacing
HF: heart failure
HFrEF: heart failure with reduced ejection fraction
HFmrEF: heart failure with mildly reduced ejection fraction
IVCDs: intraventricular conduction defects
IVMD: interventricular mechanical delay
IVS: interventricular septum
LAO: left anterior oblique
LBB: left bundle branch
LBBAP: left bundle branch area pacing
LBBB: left bundle branch block
LBBP: left bundle branch pacing
LLL: lumen-less pacing lead
LVEDD: left ventricular end-diastolic diameter
LVEDV: left ventricular end-diastolic volume
LVEF: left ventricular ejection fraction
LVESD: left ventricular end-systolic diameter
LVESV: left ventricular end-systolic volume
LVSP: left ventricular septal pacing
MRI: magnetic resonance imaging
NS-LBBP: non-selective left bundle branch pacing
NT-proBNP: N-terminal pro-brain natriuretic peptide
NYHA: New York Heart Association
pLVAT: stimulus-to-peak left ventricular activation time
PSD: peak strain dispersion
QRSd: QRS duration
RAO: right anterior oblique
RBB: right bundle branch
RBBB: right bundle branch block
RVP: right ventricular pacing
SDL: stylet-driven pacing lead
S-LBBP: selective left bundle branch pacing.
